# Systematic investigation and comparison of diagnostic methods in implant-related infections and infectious non-unions in trauma surgery– results of a prospective study

**DOI:** 10.1007/s00068-025-02900-z

**Published:** 2025-06-27

**Authors:** Robin Otchwemah, Rolf Lefering, Benedikt Marche, Thorsten Tjardes, Andreas Friedrich Wendel, Veronika Weichert, Marcel Dudda, Eva Steinhausen

**Affiliations:** 1 Institute of Hygiene, Cologne Merheim Medical Centre, University Hospital of Witten/Herdecke, Cologne, Germany; 2 Division of Hygiene and Environmental Medicine, Department of Human Medicine, Faculty of Health, Witten/Herdecke University, Witten, Germany; 3 Institute for Research in Operative Medicine (IFOM), University of Witten/Herdecke, Cologne, Germany; 4 Department of Orthopedic and Trauma Surgery Cologne-Merheim, Faculty of Health, University of Witten/Herdecke, Cologne, Germany; 5 Department for Traumatology and Orthopedics, Bundeswehr Hospital Berlin, Berlin, Germany; 6 Department of Trauma, Hand and Reconstructive Surgery, University Hospital Essen, University of Duisburg-Essen, Essen, Germany; 7 Department of Orthopedic and Trauma Surgery, BG Klinikum Duisburg, University of Duisburg-Essen, Duisburg, Germany

**Keywords:** Non-union, Low-grade-infection, Sonication, Implant-related infection, Fracture related infection

## Abstract

**Purpose:**

Alongside surgical therapy, the successful treatment of infection-related nonunion depends on the accurate identification of pathogens so as to ensure targeted anti-infective treatment. The available diagnostic methods continue to exhibit low sensitivity. The aim of the study was to determine the most sensitive method for the accurate diagnosis of infection-related nonunion by comparing sonication with other diagnostic procedures.

**Methods:**

In a prospective comparative study, 100 patients with nonunions (study group, SG) and 100 patients with planned metal removal (control group, CG) were compared. The diagnostic methods employed included standard culture, enrichment in blood culture bottles after tissue homogenization, sonication with and without membrane filtration, and histopathology. Sensitivity, specificity, and predictive values were calculated.

**Results:**

In 133 patients (SG *n* = 73; CG *n* = 60), at least one sample was tested positive for a bacterium. Infection was diagnosed in 72 patients (SG *n* = 45; CG *n* = 27). Coagulase-negative staphylococci and *Cutibacterium acnes* were the most frequently detected pathogens. Sonication achieved the highest sensitivity (80.6%) for detecting an infection but there was no significant difference as compared to the other methods. The combination of standard culture and sonication yielded the greatest overall sensitivity (97.2%), whereas the combination of tissue homogenization and histology achieved the best results for specificity (89.1%) and predictive values (82.3%).

**Conclusion:**

A high rate of pathogen detection was observed in clinically inapparent nonunions. Sonication was not clearly superior in our setting. In general, the combination of several diagnostic methods provided the most informative results. Therefore, sonication and tissue homogenization can both be considered useful additions to standard cultures.

## Background

Implant-related infections and nonunions are a significant medical and economic problem. Early infections are usually accompanied by clear infection-specific symptoms and elevated laboratory inflammatory markers, making them relatively easy to diagnose. However, late infections are often caused by less virulent organisms and may present in a subtle or even inapparent manner (“low-grade infections”) on clinical examination and in laboratory testing. The only “symptoms” may be chronic pain or the development of a nonunion.

The successful treatment of nonunions depends not only on the reliable differentiation between aseptic and septic nonunions but also on the identification of pathogens in septic nonunions. This is the only way to ensure the success of targeted antibiotic therapy [1]. In cases where the pathogen is not identified, inadequate antibiotic treatment can lead to the persistence of pathogens, to antibiotic resistance, and to continued fracture nonunion. Despite all medical advances, however, there is still no reliable method for the diagnosis of low-grade infections or definitive differentiation between septic and aseptic nonunions [2, 3].

The diagnostic gold standard for septic nonunions remains pathogen detection from tissue samples and/or a positive histology [1, 2, 4]. However, histological identification of osteomyelitis is insufficient by itself to enable the selection of effective targeted antibiotic therapy. In clinical practice, the available diagnostic methods and the method for obtaining samples are characterized by low standardization and low sensitivity. False-negative results after sampling have been reported in up to 30–40% of cases [5, 6]. Since bacteria may reside in biofilms, these pathogens are often undetectable by conventional culture methods.

In recent years, there has been increasing interest in the preparation of explanted implants for microbiological examination using the sonication method. In particular, sensitivity has been reported to be higher for biofilm-forming pathogens and mixed infections compared to conventional methods [7, 8]. Sonication has already established its place in arthroplasty. Data concerning implant-related infections and infection-related nonunions are sparse and contradictory [9–15]. There is a lack of systematic investigation of the methodology.

Other diagnostic methods that could play a role in infection diagnostics include the enrichment of tissue samples in blood culture bottles after tissue homogenization as well as molecular biological techniques such as polymerase chain reaction (PCR).

Depending on the study and diagnostic technique employed, septic nonunion rates of up to 88% have been reported [16]. In our own patient cohort, cultures detected bacteria in tissue samples taken from supposedly aseptic nonunions in 44% of the cases [17]. Based on these results, it must be assumed that low-grade infections are much more common in nonunions than previously thought.

To avoid over- or undertreatment in nonunions, a standardized approach based on systematically investigated diagnostic methods is needed. Given that molecular methods such as PCR require significant additional resources and expertise, the initial goal was to systematically investigate more widely applicable and cost-effective microbiological methods.

The aim of the study was to determine the most sensitive method for the early and accurate diagnosis of septic nonunions by comparing sonication with other diagnostic methods, with the goal of enabling targeted therapy and preventing undertreatment.

Based on the available evidence, we formulated the hypotheses that sonication is superior to traditional culture methods for the detection of low-grade infections in nonunions with respect to sensitivity and specificity and that sonication is not inferior to other diagnostic methods.

## Materials and methods

The diagnostic study was conducted prospectively and comparatively at two study centers (BG Clinic Duisburg gGmbH; Cologne Merheim Medical Centre, University Hospital of Witten/Herdecke, Cologne, Germany).

### Patient cohort

In accordance with the power analysis (see below), recruitment of 100 patients each for the study and control groups was planned. The study group (SG) included patients with nonunions of the lower extremity who had no known history of osteomyelitis and no signs of acute infection. Patients undergoing metal removal after an uncomplicated healing process of a lower extremity fracture were included in the control group (CG).Additional inclusion criteria for both groups included surgical revision planned independently of the study (SG) or metal removal planned independently of the study (CG), patient age of at least 18 years, and written consent from the patients. Exclusion criteria for both groups were a known history of osteomyelitis/infection, clinical signs of infection at the time of the planned surgery, malignant disease, chronic inflammatory disease of other origin, intravenous drug abuse, pregnancy, and participation in another intervention study.

Nonunion type and location, epidemiological data, and pre-, peri-, and postoperative progress were extracted from the digital patient records and evaluated.

“Nonunions” were defined as fractures that had not consolidated after at least six months and would not heal without further intervention [18].

Nonunions and metal implant removal sites were both classified as aseptic, septic, and highly suspected of infection based on the criteria developed by Metsemakers et al. [19]. The presence of a nonunion was considered a suggestive criterion for infection. In the CG, cases with bacterial detection in only one sample, and no further suggestive criteria of infection according to Metsemakers [19] were classified as aseptic.

Because our focus was on diagnostic procedures, evaluation of treatment decisions was not standardized or systematically evaluated. In cases of septic nonunions or metal implant removal sites, the attending surgeon determined the further treatment steps.

### Diagnostic procedures

Removed metal implants and tissue samples were diagnostically processed using four approaches:

1. Metal implants: Sonication with and without membrane filtration.
2. Tissue samples:

- Standard tissue sample cultures.
- Enrichment in blood culture bottles after tissue homogenization.
- Histopathological examination.

**Fig. 1:**
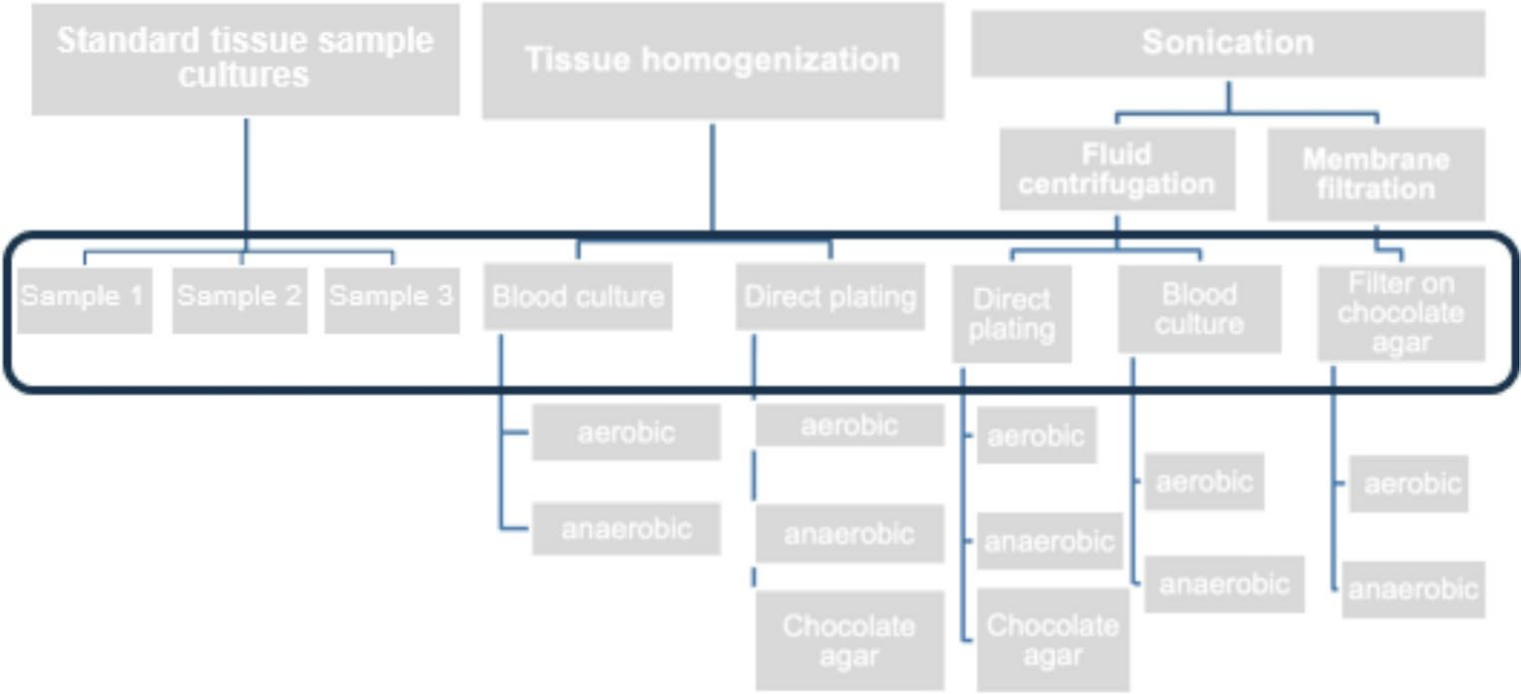
Microbiological approach with 8 samples per patient (black box)

Tissue samples were taken by trained staff and transported to the designated microbiology and pathology laboratories within 4 h. All culture media were incubated for up to 14 days at 36 °C ± 1 °C. Bacterial growth of each morphology was quantified as colony forming units (CFU). Identification and antimicrobial susceptibility testing were carried out using standard microbiological procedures, including the Vitek 2 system (bioMérieux, Marcy l’Etoile, France) and matrix-assisted laser desorption ionization-time of flight mass spectrometry (MALDI-ToF, Brucker Daltonics, Bremen, Germany).

### Sonication of metal implants

#### Validation

The sonication method was validated before routine clinical use. Titanium test bodies (KLS Martin, Germany) were inoculated with 100 µl of a bacterial suspension of a defined microbial load and quantity (10-fold dilution series, from 10^1^ to 10^9^ CFU/test body including a negative control) in triplicates. After a 2-hour drying step at room temperature to promote bacterial adhesion, the test bodies were subsequently subjected to sonication (standard procedure see below). The CFU per test body were determined using direct plating and membrane filtration on blood agar (100 µl and 100 ml of sonicate fluid, respectively) and compared to the CFU/test body applied. From this a reduction factor was determined, which represents in powers of ten the factor by which the initial bacteria count differs from the bacterial count at the end of the examination process. The investigations were conducted with *Staphylococcus epidermidis* ATCC 12,228 and *Pseudomonas aeruginosa* ATCC 9057. The process was considered to be sensitive and reliable, with average reduction factors of 0.5 for *S. epidermidis* and 1.3 for *P. aeruginosa* and with no occurrence of bacterial contamination.

#### Implant preparation

The metal implants were removed under aseptic conditions in the operating room (OR). The explant surgeon directly transferred all explanted parts into one sterile vessel (Thermo Scientific Nalgene^®^ bottle, USA) under aseptic precautions. The vessel was filled with just enough sterile Ringer’s solution to completely cover the implant with fluid but with a minimum of 300 ml and sealed with a sterile screw cap.

In the laboratory, the vessel was shaken manually for 30 s. Subsequently the sample vessels were sonicated with a frequency of 40 kHz in an ultrasonic bath (BactoSonic, Bandelin electronic GmbH & Co. KG, Germany) for 1 min at 100% intensity (= 200 W nominal output). The BactoSonic was prefilled with 100 ml of Trickopur R33 (Fa. Trick-Comp, Germany) and topped up with water. After sonication the surface of the vessels was dried, disinfected with terralin liquid (Fa. Schülke, Germany) on the outside and finally mixed manually for 30 s again. The largest possible volume of sonication fluid was used for further testing.

Up to 50 ml of sonication fluid was centrifuged for 15 min at 1,500 x g. After discarding the supernatant, the pellet was resuspended in 500 µl of NaCl 0.9%. Subsequently 100 µl of the resuspension was plated on the media (Fig. 1). The remaining concentrated fluid was filled with NaCl 0.9% to a volume of 5 ml, and 2 ml of each sample was inoculated into aerobic and anaerobic blood culture bottles (BacT/ALERT FA/FN plus, bioMeriéux, Durham, USA).

Membrane filtration volumes below 100 ml were filled with NaCl 0.9% up to 100 ml. Two times 50 ml of sonication fluid was filtered. The filters were applied to chocolate agar plates and incubated under aerobic and anaerobic conditions.

### Tissue samples

In the SG (*n* = 100) five tissue samples were taken from proximal, distal, and intermediate nonunion sites or the medullary cavity (bone and soft tissue) for microbiological and histopathological evaluation. In the CG (*n* = 100) the same number of samples were collected from the implant site and the healed fracture site or the medullary cavity (soft tissue or bony debris) respectively. In the OR the specimen was put in a sterile vessel and NaCl 0.9% was added to prevent the tissues from drying. Samples were then transported to the lab for further evaluation.

Three tissue samples were inoculated in liquid media without further processing of the tissue. This approach is referred to as “standard tissue sample culture.”

After its weight was determined, one tissue sample per patient was transferred to a mixing vessel containing sterile steel beads and 5 ml of NaCl 0.9% (ProbeAX, Axonbiotech GmbH, Germany) for homogenization. Subsequently, another 1 ml of NaCl 0.9% was added and the sample was homogenized in the ULTRA-TURRAX^®^ Tube Drive Workstation (IKA Werk GmbH, Germany) at maximum level for one minute. Depending on the weight and texture of the sample, the process was extended. Subsequently, 100 µl of homogenized tissue was plated out on two blood agar media (anaerobic and aerobic incubation) and chocolate agar (incubated in 5–10% CO_2_). Additionally, aerobic and anaerobic blood culture bottles were incubated with 2 ml of fluid each.

In cases of positive microbial detection, an evaluation of the microbial load and identity was conducted. For better evaluation, pathogens were grouped (*Staphylococcus aureus*, coagulase-negative staphylococci (CoNS), *Cutibacterium* acnes, other gram-positive and gram-negative pathogens).

### Histopathological examination

One tissue sample per patient was taken from the implant membrane or the nonunion site and fixated in formaldehyde in the OR for histopathological evaluation. Tissues were decalcified if necessary and further processed using HE and EvG staining and the PAS reaction. Afterwards, the samples were assessed by means of the histopathological osteomyelitis evaluation score (HOES) [20], or in the case of implant membranes, using the method proposed by Morawietz et al. [21].

### Statistics

Sample size calculation: The assumption was a septic nonunion incidence of 44%. This corresponds, on the one hand, to the infection rate observed in our own patient cohort, and on the other hand, to the mean value reported in other studies. With a presumed error probability of α = 0.05 and a power of 90%, a 95% confidence interval of ± 0.33 SD (standard deviation) can be achieved with 100 patients in both the study and control groups. The Wilcoxon test was applied (dependent samples, paired design).

For the determination of sensitivity (correctly identified infected patients by each diagnostic method), specificity (correctly identified non infected patients by each diagnostic method), positive predictive value (probability that patients with bacterial detection are actually suffering from a fracture-related infection (FRI)), negative predictive value (probability that patients without bacterial detection are actually not suffering from a FRI) and test accuracy (proportion of correctly classified patients (both true FRI and true non-FRI) among the tested individuals) only patients with confirmed FRI from the SG and the CG were considered infected. Patients with suspected FRI and without FRI were considered non-infected.

#### Statistical analysis

All data were pseudonymized in a database and anonymized before evaluation. The groups (SG vs. CG and FRI vs. aseptic nonunion) were compared using Fisher’s exact test for binary data, and the Mann-Whitney U test for continuous data. For descriptive analysis, the median was provided in addition to the mean and standard deviation (SD) in case of a skewed distribution. A p-value < 0.05 was considered statistically significant. Positive and negative predictive values, sensitivity, and specificity with 95% confidence intervals were calculated for each diagnostic procedure. Statistical analysis was performed using SPSS (Version 24, IBM Inc., Armonk, NY, USA).

## Results

A total of 200 patients were prospectively included. A complete dataset was available for all patients.

### Epidemiology/Preoperative findings

SG and CG were comparable in terms of epidemiological data and pre-existing conditions.

The tibia was the most commonly affected bone in both groups, with the distal metaphysis being predominantly involved. In the SG, atrophic and hypotrophic nonunions were more common.

Differences between the groups were observed in the preoperative clinical presentation, with significantly more frequent occurrences of weight-bearing insufficiency, swelling, and implant loosening in the SG, as was expected based on the inclusion and exclusion criteria. Laboratory infection parameters (leukocytes, CRP) were significantly higher in the SG compared to the CG, although these values did not reach levels indicative of infection (Table 1).

**Table 1:** Epidemiology and preoperative findings

	Overall *n* = 200	Study Group (non-union) *n* = 100	Control Goup (Implant removal) *n* = 100	*p*-value
Epidemiology/Pre-existing Conditions/Risk Factors				
Gender (male)	112	58	54	0.67
Age (y) (Mean ± SD)	48.7 ± 15.1	50.0 ± 14.7	47.4 ± 15.4	0.23
BMI* (Mean ± SD)	27.1 ± 5.2	28.0 ± 5.7	26.3 ± 4.6	**0.02**
Obesity (n)	51	31	20	0.10
Nicotine use (n)	51	28	23	0.52
Alcohol use (n)	2	2	0	0.50
Diabetes mellitus (n)	4	3	1	0.62
pAOD* (n)	3	3	0	0.25
CAD* (n)	7	4	3	0.70
Preoperative Findings				
Femur localization (n)	54	40	14	**< 0.001**
Tibia localization (n)	96	48	48	1.00
Non-union (atrophic/hypotrophic/hypertrophic) (n)		28/58/14	-	-
Fracture type (initially open) (n)	36	23	13	0.10
> 2 prior surgeries (n)	20	14	6	**< 0.001**
Flap surgery (n)	2	1	1	1.00
Spongiosa/Bone replacement (n)	16/ 9	13/ 7	3/ 2	**0.016**/0.17
Low-dose pulsed ultrasound (n)	13	13	0	**< 0.001**
Preoperative Clinical Findings				
Edema/Swelling (n)	28	22	6	**0.002**
Load insufficiency (n)	112	95	17	**< 0.001**
Implant loosening (n)	34	31	3	**< 0.001**
Leukocytes (10^3^/µl)	7.35	7.7	7.0	**0.021**
CRP* (mg/dl)	0.37	0.46	0.27	**0.003**

*BMI, Body Mass Index; *pAOD,* Peripheral arterial occlusive disease; *CAD,* Coronary artery disease; *CRP,* C-reactive Protein

### Microbiological and histopathological results

**Fig. 2:**
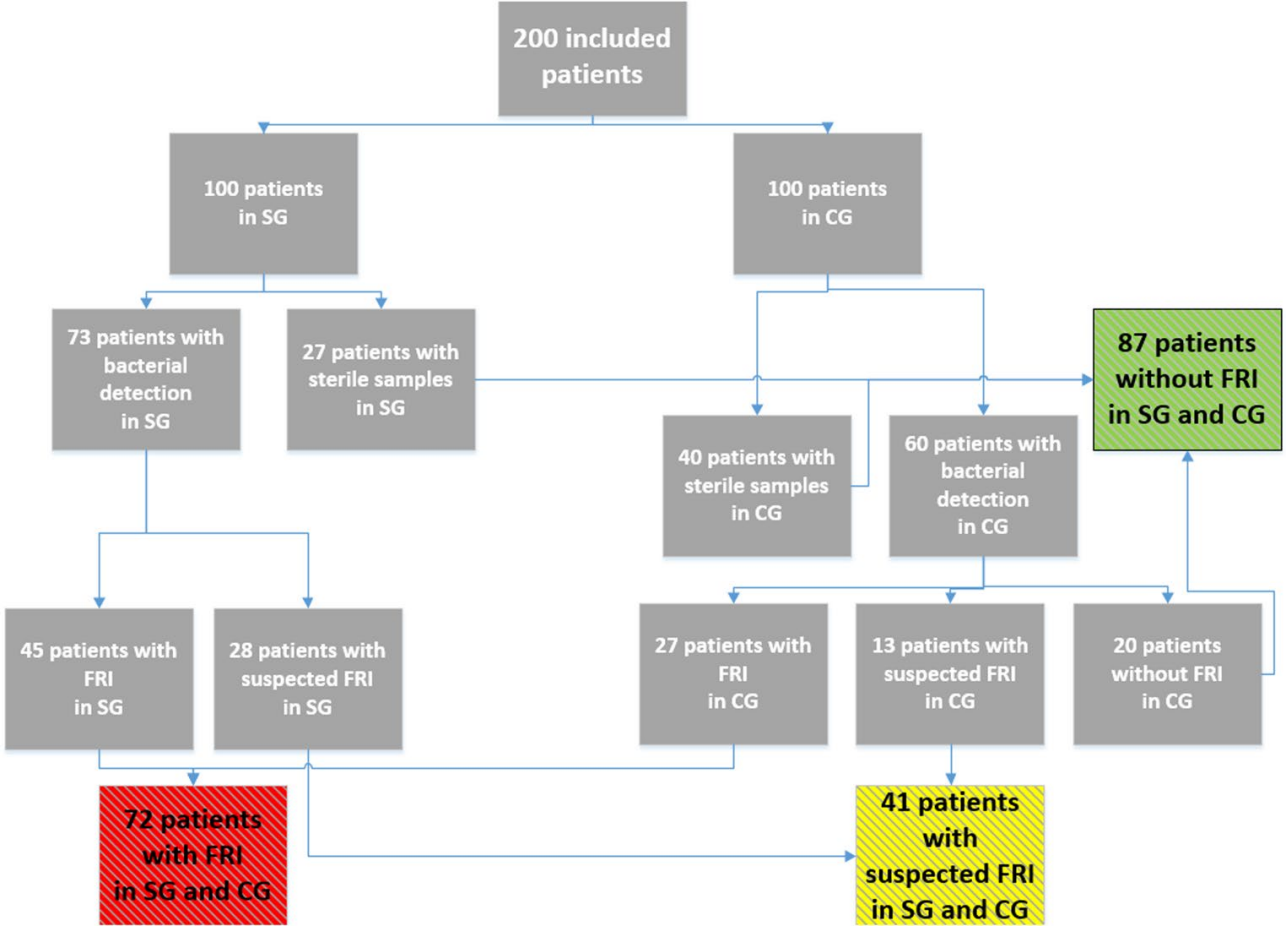
Distribution of the Patients regarding bacterial detections and FRI diagnosis. SG study group, CG control group, FRI fracture related infection

In 67 patients (SG *n* = 27, CG *n* = 40), all microbiological investigations were sterile. In 133 patients (SG *n* = 73, CG *n* = 60, *p* = 0.07), at least one microbiological sample showed a pathogen (Fig. 2).

A total of 601 out of 1,600 (37.6%) samples were positive. Of these, 305 out of 600 (50.8%) were positive in sonication, 110 out of 600 (18.3%) in standard culture, and 186 out of 400 (46.5%) in enrichment after tissue homogenization. Of the 601 positive findings, 224 applied to the CG (37.3%) and 377 to the SG (62.7%).

Sonication yielded the greatest number of positive results in 91 patients overall (SG *n* = 51, CG *n* = 40, *p* = 0.16). Standard culture tests detected a pathogen in at least one sample in 87 patients (SG *n* = 48, CG *n* = 39, *p* = 0.25). Enrichment after tissue homogenization showed a positive result in 67 patients, and was the only diagnostic method showing a significant difference between the two groups (SG *n* = 41, CG *n* = 26, *p* = 0.04).

In 56 out of 73 patients (77%) in the SG, pathogens (not necessarily identical, indistinguishable pathogens) were detected in more than one sample, with an average of 3.8 positive samples per patient. In the CG, 43 out of 60 patients (72%) had more than one positive sample (not necessarily identical, indistinguishable pathogens), with an average of 2.2 positive samples per patient. The number of positive samples differed significantly between the groups (*p* = 0.007).

Histopathological examination showed signs of osteomyelitis in 24 patients, five of whom were in the CG. Only one patient had a positive histology despite sterile results in all microbiological investigations. All other patients with positive histology also had at least one sample with a positive microbiological result.

### Detected pathogens

As expected, there were significantly more bacterial detections in the FRI than in the “no infection” group and a trend towards more detections in the SG compared to the CG. Low-virulent pathogens were predominant. Coagulase-negative staphylococci (CoNS) were the most frequently detected pathogens in both groups and across all diagnostic methods (*n* = 407 positive tests, 67.7%), with no significant differences between the two groups. *Cutibacterium acnes* was also frequently detected by all methods (*n* = 100 positive tests, 16.6%), with significantly more cases in the SG than in the CG (SG 31% vs. CG 14%, *p* = 0.006). (Table 2).

**Table 2:** Frequency and distribution of detected pathogens by patient group and diagnostic method

	Study Group (SG) (*n* = 100)*	Control Group (CG) (*n* = 100)*	*p*	FRI (Infection) (*n* = 72)*	No Infection (*n* = 87)*	*p*-value	Suspected FRI (*n* = 41)*	Sonication (*n* = 200)*	Standardculture (*n* = 200)*	Tissue homogeni-zation (*n* = 200)*
*CoNS*	60	47	0.89	64	16	**< 0.001**	27	77	74	60
*Cutibacterium acnes*	31	14	**0.006**	27	6	**< 0.001**	12	34	32	23
*Staph. aureus*	0	3	0.094	3	0	0.091	0	3	2	3
*Other gram-positive pathogens*	17	22	0.131	16	9	**0.05**	14	27	22	19
*Other gram-negative pathogens*	6	5	1.0	6	2	0.142	3	8	7	6

CoNS, Coagulase-negative staphylococci

*number of patients

### Fracture-related infection (FRI)

FRI, based on the criteria of Metsemakers et al. [19], was diagnosed in 72 patients (SG *n* = 45 CG *n* = 27). The difference between the two groups was significant (*p* = 0.012).

Patients with FRI had significantly more preoperative clinical signs of swelling (75% vs. 25%) or warmth (66.7% vs. 33.3%).

Correlating with the microbiological findings, patients with FRI showed significantly more radiological signs of osteolysis, sequestra, periosteal reactions, and implant loosening.

In patients with confirmed FRI, coagulase-negative staphylococci and *Cutibacterium acnes* were the most frequently detected pathogens (Table 3).

**Table 3:** Frequency and distribution of detected pathogens in FRI by patient group and diagnostic method

	Overall (*n* = 72)*	Study Group (*n* = 45)*	Control Group (*n* = 27)*	*p*-value	Sonication (*n* = 58)*	Standard culture (*n* = 57)*	Tissue homogenization (*n* = 53)*
*CoNS*	64	40	24	1.00	53	54	50
*Cutibacterium acnes*	27	22	5	0.012	25	23	21
*Staph. aureus*	3	0	3	**0.049**	3	2	3
*Other gram-positive pathogens*	16	9	7	0.572	12	10	10
*Other gram-negative pathogens*	6	4	2	0.826	2	2	2

* number of patients with FRI

CoNS, Coagulase-negative staphylococci

Overall, of the 72 patients with confirmed FRI, 23 patients had negative results in sonication, 19 patients had negative results in blood cultures after tissue homogenization, and 17 patients had negative results in standard cultures.

Only 23 out of 72 patients with FRI (31.9%) had positive results in all microbiological testing methods. No patients with FRI were found to have a positive result only in tissue homogenization or only in sonication (Table 4).

**Table 4:** Correlation between FRI and positive findings by diagnostic method

Positive Diagnostic Methods in FRI	Number of patients
Sonication + Standard culture + Tissue homogenization	23 (31.9%)
Sonication + Tissue homogenization	17 (23.6%)
Sonication + Standard culture	9 (12.5%)
Standard culture + Tissue homogenization	13 (18.1%)
Standard culture	10 (13.9%)
**Overall**	**72 (100%)**

Sensitivity and negative predictive value did not differ significantly between the individual diagnostic methods. Regarding specificity and positive predictive value, the tissue homogenization method achieved the highest values (Table 5). We did not find a difference between the sensitivity of sonication regarding extra- (80.5%) or intramedullary (80.6%) implants.

By combining different diagnostic methods, it was possible to improve sensitivity. The combination of standard methods and sonication achieved the highest sensitivity at 97.2%, whereas the combination of tissue homogenization and histology provided the best results for specificity, positive and negative predictive values (Table 5).

In patients with confirmed FRI, the proportion of pathogen detection in sonication with direct application of the sonication fluid without prior membrane filtration was 50% (95% CI: 35%, 66%), in sonication with filtration it was 67% (95% CI: 56%, 77%), and for both methods combined it was 82.6% (95% CI: 72%, 94%). Thus, the combination of membrane filtration and direct application of the sonication fluid resulted in more frequent pathogen detection in infected nonunions in our setting than direct application alone.

**Table 5:** Sensitivity, specificity, positive and negative predictive values, accuracy by diagnostic method and their combinations (Based on patients *n* = 200)

	Sensitivity (%)	Specificity (%)	Positive predictive value (%)	Negative predictive value (%)	Accuracy (%)	Confidence intervall (of accuracy)
Standard culture	79.2	76.6	65.5	86.7	77.5	71.7–83.3
Sonication *(overall)*	80.6	74.2	63.7	87.2	76.5	70.6–82.4
Sonication conventional	56.9	85.9	69.5	78.0	75.5	69.5–81.5
Sonication with membrane filtration	79.2	78.9	67,9	85,7	78.0	72.3–83.7
Tissue homogenization	73.6	89.1	79.1	85.7	84.5	79.5–89.5
Histology	33.3	100	100	72.7	76.0	70.1–81.9
Standard culture and histology	88.9	76.6	68.1	92.5	81.0	75.6–86.4
Sonication and histology	90.3	74.2	66.3	93.1	80.0	74.5–85.5
Tissue homogenization and histology	90.3	89.1	82.3	94.2	89.5	85.3–93.7
Standard culture and sonication	97.2	57.8	56.5	97.4	72.0	65.8–78.2
Standard culture and tissue homogenization	93.1	68.0	62.0	94.6	77.0	71.2–82.8

### CFU quantification (sonication)

**Fig. 3:**
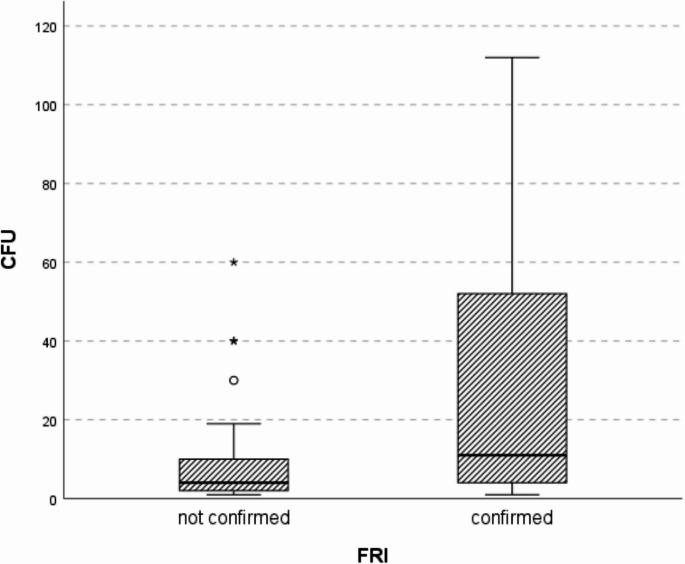
Boxplot of colony forming units (CFU) quantification in sonication, depending on confirmed vs. unconfirmed fracture related infections (FRI)

When differentiating between confirmed infection and suspected (unconfirmed) infection, significant differences were found in the number of CFU (Fig. 3).

In the “FRI confirmed” group, five values were found to be above the depicted range (> 200).

In tissue homogenization, pathogen detection was achieved in a total of 67 patients. The average weight of these positive tissue samples was 1.6 g. The average weight of the negative tissue samples was 0.9 g, which was significantly lower than the weight of the samples with pathogen detection (*p* < 0.001). In this context, it is worth noting that the average weight of the collected tissue samples in the study group (1.9 g) was significantly higher than in the control group (0.4 g; *p* < 0.001). When considering the weights exclusively within each group, the control group showed a significantly higher weight (0.5 g vs. 0.3 g; *p* = 0.024) for positive samples, and in the study group, there was a trend toward the same relationship (2.3 g vs. 1.5 g; *p* = 0.19).

## Discussion

The diagnosis of septic nonunion is challenging due to its heterogeneous appearance, especially low-grade infections may be clinically inapparent [22, 23]. Therapeutically, septic nonunions require a specific interdisciplinary approach. Pathogen identification forms the basis for diagnosis and also for effective anti-infective therapy [22, 24]. In the past, several microbiological methods have been used for diagnosis, but no single method exists that can reliably identify low-grade infections. The aim of our study was to determine the optimal diagnostic procedure for the early and accurate detection of low-grade infections in nonunions. We examined 100 patients with nonunions (study group) and 100 patients with metal removal after uncomplicated fracture healing (control group).

Laboratory infection markers were significantly higher in the study group compared to the control group, but they failed to reach values suggestive of infection. This corresponds to the findings from other studies, where laboratory infection parameters were also found to have limited diagnostic value [15, 23, 25].

FRI were diagnosed in 45% of patients. This relatively high rate is consistent with the magnitude of unexpected positive microbiological findings in nonunion cases reported in other studies [17, 26, 27] and underscores the importance of targeted diagnostics, even in the absence of clinical signs of infection.

The majority of bacterial detections were achieved using sonication, followed by standard culture. Tissue homogenization yielded the fewest positive results, but in contrast to the previously mentioned methods, it demonstrated a significantly higher detection rate in the study group than in the control group. This pattern is reflected in the relatively high values for tissue homogenization regarding specificity and positive and negative predictive values, suggesting that this method should be given further attention. Other authors have also shown that tissue homogenization can rapidly, reliably, and cost-effectively detect slow-growing bacteria such as *Cutibacterium acnes* [28–31].

Standard culture, still the gold standard [32], achieved higher sensitivity (79.2%) than tissue homogenization, but lower values for specificity and positive predictive value, which is in line with the findings of other studies [6, 25, 33, 34]. A possible reason for the diminished accuracy of standard culture results could be the ongoing need for standardization of the sample collection process. Hellebrekers et al. demonstrated that standardizing sample collection leads to higher quality results [35]. Onsea et al. [32] also emphasized the need to standardize sample collection in order to improve the quality of study results. Nevertheless, a large portion of the standardization steps proposed by Hellebrekers were followed in our study. The only step in Hellebrekers’ recommendations not implemented in our study was the change of surgical instruments for each sample collection. Furthermore, all laboratory steps were standardized and validated.

High values for the measured test quality characteristics were achieved with the combination of tissue homogenization and histology. It is important to note that a positive histology under the applied study criteria leads directly to the diagnosis of septic nonunion in our setting (specificity 100%). It should also be noted that, with the exception of one case, a positive histology always accompanied bacterial pathogen detection in the microbiological samples. In 13 patients, the infection would have been diagnosed without positive histology. However, in 10 patients without the positive histological result, there would only have been suspicion for infection. The sensitivity of histopathological examination alone was low in our study (33.3%). Depending on the method used, very low sensitivities for histology have been described in the current literature as well [26, 33, 36]. Therefore, the main role for histopathological examination is in combination with microbiological methods, in part because a positive histology cannot provide antibacterial susceptibility testing in the absence of pathogen detection.

Sonication achieved the highest sensitivity as a single diagnostic method. The combination of standard culture and sonication achieved the highest overall sensitivity, though at the cost of specificity and predictive values.

Sonication has also been investigated in other studies for bacterial identification in nonunions. Trenkwalder et al. identified sonication as the most sensitive single method, depending on the chosen cut-off value (≥ 1.36 CFU/ml sonication fluid) [36]. The CFU/ml value in the study by Trenkwalder et al. was a factor of ten lower than the values we measured in our study. We found an average of 10.5 CFU/ml sonication fluid (+/−14.0) in patients with pathogen detection where infection could not ultimately be confirmed according to the consensus criteria. Trampuz et al. established a threshold of 50 CFU/ml sonication fluid as the benchmark for the diagnosis of prosthetic joint infections [37]. Our findings underscore the need for further investigation to determine a universally applicable cut-off value. Sonication, which has already been established for the diagnosis of prosthetic infection, would appear to offer advantages in the context of low-grade infections in nonunions/FRI, particularly through biofilm disruption [38]. In terms of specificity and predictive values, however, our study did not show any clear superiority of sonication over other methods.

It was also observed that only 23 out of 72 patients with diagnosed FRI (31.9%) had positive findings on all microbiological tests. This indicates that in our setting, a combination of various diagnostic methods was generally required for a sufficiently reliable diagnosis, which in turn increased the likelihood of pathogen detection.

As expected, pathogens were detected significantly more frequently in the study group. However, pathogen detection also occurred regularly in the control group. Bacterial sample contamination cannot be excluded as a potential cause for this finding, although in the course of the study preparation, laboratory validation of sonication was performed without detecting any contamination. The relatively high number of affected patients in the control group along with the reports that other authors have also detected pathogens in control groups from healed fractures, suggest the real presence of bacteria [36].

Since no systematic clinical follow-up of the enrolled patients was conducted, the clinical relevance of the pathogen findings could not be conclusively determined in this study. However, international consensus criteria for the definition of infection were applied to ensure valid infection diagnoses. As part of preliminary follow-up of 71 patients from the study group over a period of at least one year, 35 patients were diagnosed with an FRI, and 21 were classified as suspected FRI in analyses to date. Postoperative antibiotic therapy was administered to 69% (*n* = 24) of the patients with FRI and to 24% (*n* = 5) of the patients with suspected FRI. After one year, 89% (*n* = 31) of nonunions with FRI and 95% (*n* = 20) of nonunions with suspected FRI had achieved bony consolidation, including 11 FRI patients (31%) who had not received postoperative antibiotic treatment. Among the 27 patients in the control group diagnosed with FRI, only one developed a symptomatic postoperative wound infection (with detection of *Staphylococcus aureus*) necessitating surgical revision and systemic antibiotic therapy. No complications occurred in any other control group patients despite a diagnosis of FRI and the absence of further antibiotic treatment. These findings suggest that in certain cases, bacterial detection does not necessarily require antibiotic therapy to achieve soft tissue healing or bone consolidation. Further studies are needed to identify factors and conditions that promote bone consolidation despite the presence of bacteria. The goal would be to determine whether an FRI requiring treatment is present based on these identified conditions and ideally to establish cut-off values for pathogen load.

As is consistent with low-grade infections, our study predominantly detected low-virulence pathogens were detected. Coagulase-negative staphylococci were the most frequently cultured organisms. *Cutibacterium acnes* was detected significantly more often in the study group than in the control group and the FRI group than in the non-infection group. This finding, despite its low pathogenicity, could indicate the potential relevance of *Cutibacterium acnes* for the identification of low-grade infections in nonunions. Taking into account only confirmed FRI patients, *Staphylococcus aureus* was detected more often in the CG. However, the absolute number of patients was low, which calls the significance of this finding into question. Moreover, other gram-positive pathogens were significantly more common in the group with confirmed FRIs when compared to the group without infection. However, it should be considered that this group is very heterogeneous in terms of the pathogens detected and also that individual bacterial species occurred in only small numbers. Therefore, this finding should be interpreted with caution.

When considering the weights of the tissue samples taken for homogenization within the groups, it became evident that the samples from the infected group with pathogen detection were significantly heavier than those without. A similar trend was observed in the non-infected group. This underscores the importance of obtaining as large a sample of tissue as possible during clinical investigation when searching for pathogens.

### Limitations

Our study has several limitations. Besides the absence of clinical follow-up of the patients already described, PCR techniques were not employed to analyze the collected material. However, this method has not been standardized in the context of nonunions and, accordingly, yields inconsistent results. For example, Palmer et al. detected bacteria in 88% (30/34) of clinically aseptic nonunions using PCR, whereas cultural methods only identified bacteria in 24% of cases. Biofilm formation is considered a major reason for such significantly differing results. By contrast, Gille et al. detected bacteria in only 8.7% of supposedly aseptic tibial nonunions (*n* = 23) using PCR [2].

## Conclusion

In summary, our study revealed a relatively high rate of pathogen detection in clinically inapparent nonunions, indicative of a low-grade infection. Sonication was the microbiological diagnostic method with the greatest sensitivity, but no significant differences in sensitivity were found between the individual methods. Overall, combining multiple diagnostic methods provided the most meaningful results. Both sonication and tissue homogenization can be considered as useful adjuncts to the gold standard (standard culture) but cannot yet be described as unequivocally superior. The quality of sample collection plays a crucial role in all diagnostic methods and should be standardized. The clinical relevance of the detected low-grade infections should be the subject of future research.

## Data Availability

No datasets were generated or analysed during the current study.

## Declarations

### **Trial registration**

DRKS00014014. Date of registration: 15.02.2018
